# Metallic Ligatures in Surgery

**Published:** 1829

**Authors:** 


37. *Metallic Ligatures in Surgery.*—The 7th No. of the Amer. Journal of Med. Sciences, contains an interesting experimental paper on metallic ligatures, the use of which was first suggested by our celebrated countryman Dr. Physick. The author is Dr. Levert of Alabama. His experiments were on the carotid and femoral arteries of dogs, and seem to have been executed with care. They were made with four different kinds of wire—lead, silver, gold and platinum—all metals difficult to oxydize. After exposing the artery,the wire was carried once round it, and drawn so tight as to obstruct the circulation, when the ends were cut off, and the wound closed up, to heal by the first intention; which however did not always happen. But whether it granulated or not, he invariably found on examining the part after it had cicatrized, that the calibre of the vessel was obliterated, and the wire enclosed in a cyst of dense cellular substance. There was neither suppuration nor inflammation in its vicinity.

For the purpose of comparison he employed, in other experiments, ligatures of silk, elastic gum and grass. The wounds healed up, but he found, on examination, that the ligatures of the two former substances were surrounded by pus; those of grass were enclosed in a cyst, which, however, was moist and uneven, and did not embrace them closely. On the whole, Dr. L. concludes, that the plan of tying arteries with lead and other metals (not easily oxydated) is free from danger, and may be productive of some peculiar advantages; but that more experience and a greater number of experiments are necessary to establish this point thoroughly.

